# Oxidative stress in healthy pregnancy and preeclampsia is linked to chronic inflammation, iron status and vascular function

**DOI:** 10.1371/journal.pone.0202919

**Published:** 2018-09-11

**Authors:** Dominique Mannaerts, Ellen Faes, Paul Cos, Jacob J. Briedé, Wilfried Gyselaers, Jerome Cornette, Yury Gorbanev, Annemie Bogaerts, Marc Spaanderman, Emeline Van Craenenbroeck, Yves Jacquemyn

**Affiliations:** 1 Research Group ASTARC, Antwerp Surgical Training, Anatomy and Research Centre, University of Antwerp, Antwerp, Belgium; 2 Department of Obstetrics and Gynaecology, Antwerp University Hospital, Edegem, Belgium; 3 Laboratory for Microbiology, Parasitology and Hygiene (LMPH), University of Antwerp, Antwerp, Belgium; 4 Department of Toxicogenomics, School of Oncology and Developmental Biology (GROW), Maastricht University, Maastricht, The Netherlands; 5 Department of Obstetrics and Gynaecology, Ziekenhuis-Oost Limburg, Genk, Belgium; 6 Department of Obstetrics and Gynaecology, Erasmus M.C. Rotterdam, Rotterdam, The Netherlands; 7 Research Group PLASMANT, Department of Chemistry, University of Antwerp, Antwerp, Belgium; 8 Department of Obstetrics and Gynaecology, MUMC Maastricht University, Maastricht, The Netherlands; 9 Laboratory for Cellular and Molecular Cardiology and Department of Cardiology, Antwerp University Hospital, Edegem, Belgium; 10 Research Group Cardiovascular Diseases, Translational Pathophysiological Research, University of Antwerp, Antwerp, Belgium; University of Southampton, UNITED KINGDOM

## Abstract

**Background:**

During normal pregnancy, placental oxidative stress (OS) is present during all three trimesters and is necessary to obtain normal cell function. However, if OS reaches a certain level, pregnancy complications might arise. In preeclampsia (PE), a dangerous pregnancy specific hypertensive disorder, OS induced in the ischemic placenta causes a systemic inflammatory response and activates maternal endothelial cells. In this study, we aimed to quantify superoxide concentrations (as a measure of systemic OS) using electron paramagnetic resonance (EPR) and correlate them to markers of systemic inflammation, iron status and vascular function.

**Methods:**

Fifty-nine women with a healthy pregnancy (HP), 10 non-pregnant controls (NP) and 28 PE patients (32±3.3weeks) were included. During HP, blood samples for superoxide, neutrophil to lymphocyte ratio (NLR), mean platelet volume (MPV) and iron status were taken at 10, 25 and 39 weeks. Vascular measurements for arterial stiffness (carotid-femoral pulse wave velocity (CF-PWV), augmentation index (AIx), augmentation Pressure (AP)) and microvascular endothelial function (reactive hyperemia index (RHI)) were performed at 35 weeks. In PE, all measurements were performed at diagnosis. CMH (1-hydroxy-3-methoxycarbonyl-2,2,5,5-tetramethylpyrrolidine) was used as spin probe for EPR, since the formed CM radical corresponds to the amount of superoxide.

**Results:**

Superoxide concentration remains stable during pregnancy (p = 0.92), but is significantly higher compared to the NP controls (p<0.0001). At 25 weeks, there is a significant positive correlation between superoxide and ferritin concentration. (p = 0.04) In PE, superoxide, systemic inflammation and iron status are much higher compared to HP (all p<0.001). During HP, superoxide concentrations correlate significantly with arterial stiffness (all p<0.04), while in PE superoxide is significantly correlated to microvascular endothelial function (p = 0.03).

**Conclusions:**

During HP there is an increased but stable oxidative environment, which is correlated to ferritin concentration. If superoxide levels increase, there is an augmentation in arterial stiffness. In PE pregnancies, systemic inflammation and superoxide concentrations are higher and result in a deterioration of endothelial function. Together, these findings support the hypothesis that vascular function is directly linked to the amount of OS and that measurement of OS in combination with vascular function tests might be used in the prediction of PE.

## Introduction

Pregnancy is a state characterized by many physiological changes, which would be pathological in the non-pregnant state. Haematologically, neutrophils are increased due to augmented physiologic stress and impaired neutrophilic apoptosis during pregnancy, while lymphocytes are known to decrease during normal pregnancy, with a rise during the third trimester. Delivery is a highly stressful situation, resulting in a brisk leucocytosis. [1, 2] Thrombocytes on the other hand, become activated during pregnancy, particularly in the third trimester, and thus decrease, a condition referred to as ‘gestational thrombocytopenia’. [2] This systemic inflammatory response in pregnancy results in high amounts of circulating reactive oxygen species (ROS), produced by activated blood cells. [3] The central organ regulating pregnancy, the placenta, is a major source of ROS. During normal pregnancy, placental oxidative stress (OS) is present during all three trimesters and is necessary to obtain normal cell function, including activation of redox-sensitive transcription factors and protein kinases. [3–7] Although OS is a common necessary feature of normal pregnancy, augmented OS could give rise to different disease-states, such as preeclampsia (PE). PE is a potentially life-threatening complication of pregnancy, clinically detected after 20 weeks gestation. It affects 5% of pregnancies and is characterized by hypertension and proteinuria in mild cases, but can derail into organ damage, seizures and maternal death in severe cases. In the classic two-stage model of PE, OS induced in the ischemic placenta causes release of cytotoxic factors into the maternal circulation, stimulating the inflammatory response and activating maternal endothelial cells. [3] Increased neutrophil to lymphocyte ratio (NLR) and mean platelet volume (MPV) have been suggested as parameters of this chronic low-grade inflammation and enhanced OS. [8–10] Both systemic inflammation and OS result in the formation of ROS and reactive nitrogen species (RNS). ROS and RNS can react with nitric oxide (NO), resulting in a lower bio-availability of NO, the main player in endothelial function. The combination of superoxide and NO forms ONOO- (peroxynitrite), a harmful molecule with cell destructive effects. [11] This disturbance in endothelial homeostasis can lead to endothelial dysfunction, a condition characterized by a vasoconstrictive, pro-inflammatory and prothrombotic tendency. [12–14] In PE, OS, systemic inflammation and vascular dysfunction are obviously linked and capable of forming dangerous positive feed-forward systems. [3]

Under physiological conditions, the most common oxygen free radical in the human body is the superoxide anion radical (O2^∙-^). Superoxide concentration is increased under conditions of hypoxia, when the availability of oxygen to act as final electron acceptor in the mitochondrial respiratory chain is reduced, which results in accumulation of unpaired electrons on oxygen. [3] As a result, iron (Fe) plays a crucial catalysing role in the production of ROS via the formation of hydroxide (OH^−^) and the very reactive hydroxyl radical (HO^∙^), the main products of the Haber-Weiss and Fenton reactions. [15, 16] Literature suggests that an increased iron and ferritin concentration is linked to a higher risk of PE. [16] The profound hemodynamic changes of pregnancy can be objectified by assessing endothelial function and vascular stiffness with peripheral arterial tonometry (PAT) and applanation tonometry and they are proven to be disturbed in PE pregnancies. [9] Determination of ROS in blood during pregnancy and PE is of wide interest to specialists in perinatal medicine, but the best technique to access OS has been matter of debate. [5, 17]Therefore, we performed a study by applying electron paramagnetic/spin resonance (EPR/ESR) spectroscopy as a direct method to detect ROS by using the spin-probe CMH (hydroxy-3-methoxycarbonyl-2,2,5,5-tetramethylpyrrolidine) to detect superoxide concentrations in blood. [18–21]

Although endothelial dysfunction and vascular stiffness are proven to be present in PE, no direct correlation with markers of OS and systemic inflammation has been proven. In this study, we aim to elucidate this correlation by measuring superoxide concentrations, haematological parameters of systemic inflammation and iron concentrations in HP and PE and correlate them with vascular function measurements.

## Material and methods

### Study population

Fifty-nine women with a healthy pregnancy (HP) and 28 PE patients (gestational age 25+6 weeks—38+1 weeks (mean 32±3.3weeks)) admitted to the maternal intensive care unit were included between January 2016 and September 2017. We defined PE according to the revised ISSHP definition (2014).[22] Exclusion criteria were (gestational) diabetes, multiple pregnancies, foetal malformations, hypercholesterolemia, kidney disease, auto-immune disorders, connective tissue diseases or use of acetylsalicylic acid. HP were included in the study during their first trimester and were longitudinally followed throughout the whole pregnancy. They were free from medication and did not have a history of PE, pregnancy-induced hypertension, hypertension, cardiovascular disease or other chronic conditions. A small group of non-pregnant (NP) healthy controls (n = 10) was included to compare OS concentrations. The Research and Ethics committee of the Antwerp University Hospital approved the study protocol (Belgian number: B300201524783), and written informed consent was obtained from all subjects.

### Oxidative stress: Superoxide concentrations

EPR measurements were carried out on a Bruker EMX 1273 spectrometer equipped with an ER 4119HS high-sensitivity resonator and 12-kW power supply operating at X band frequencies. [23] The EPR analysis setting were as follows: frequency 9.86 GHz, power 50.41 mW, modulation frequency 100 kHz, modulation amplitude 1 G, sweep time 41.94 sec, time constant 40.96 msec, sweep width 50 G, number of scans 1. For the measurements, maternal blood was obtained at 9–11 weeks (mean 10+3 weeks), 24–28 weeks (mean 25+2 weeks) and at 38–41 weeks (mean 39+3weeks) in a heparin tube (BD vacutainer^®^, Canada) and transported on ice. After 15 minutes, 30μl of spin probe CMH (1-hydroxy-3-methoxycarbonyl-2,2,5,5-tetramethylpyrrolidine) was added to 30μl blood. The mixture of CMH and blood was incubated on ice and transferred into a 50 μl glass capillary (Hirschmann^®^, Germany) after 5 minutes. The sample was snap frozen and stored at -80°C until analysis. [23] The concentrations reported were obtained via double integration of the respective simulated spectra of the formed CM radical and this corresponds to the amount of superoxide that was present in the sample. The simulations were performed using a NIEHS P.E.S.T. WinSIM ver. 0.96 using the hyperfine values obtained from literature. [24] EPR calibration was performed using aqueous solutions of a stable radical 4-hydroxy-TEMPO (97%, Sigma-Aldrich, Germany) in a range of concentrations 2–200 μM as previously described. [25]

### Haematological parameters of systemic inflammation (NLR/MPV) and iron status

Maternal venous blood samples were taken for the quantification of mean platelet volume (MPV), neutrophil-lymphocyte ratio (NLR), iron, ferritin and transferrin concentrations. Peripheral blood was collected by venepuncture at standard follow-up visits: 9–11 weeks (mean 10+4 weeks), 24–28 weeks (mean 25+3 weeks), and at 38–41 weeks (mean 39+3weeks) using vacuette tubes. Ethylenediaminetetraacetic acid (EDTA) and serum samples (BD Vacutainer^®^, Canada) were analysed respectively using an ADVIA 120 Hematology System (Siemens healthcare^®^, Germany) and Dimension Vista 1500 System (Siemens healthcare^®^, Germany).

### Vascular function measurements

Vascular function measurements were taken during the third pregnancy trimester in HP and at the time of diagnosis in the PE group. Blood pressure measurements were taken using an automated blood pressure device (OMRON^®^ Intellisense, Healthcare Japan). Microvascular endothelial function was evaluated with PAT using the Endo-PAT2000^®^ (Itamar Medical, software version 3.2.4) and systemic arterial stiffness was evaluated using applanation tonometry with pulse wave analysis (PWA) and pulse wave velocity (PWV) using the Sphygmocor system^®^ (Atcor Medical, West Ryde, Australia). PWA derives augmentation pressure (AP) and augmentation index (standardized to a heart rate of 75 bpm, AIx75) from the aortic pressure waveform. While PWV measures the carotid-femoral pulse wave velocity (CF-PWV), the gold standard for assessing aortic stiffness. PAT is an operator-independent and highly reproducible technique. [26] The system uses pneumatic finger probes which assess digital volume changes accompanying pulse waves. Relative ischaemia was induced by inflation of a blood-pressure cuff to suprasystolic pressure on the forearm of the patient for 5 minutes, after which the pressure was released and reactive hyperaemia was measured. The increased shear stress induced by reactive hyperaemia, increases endothelial NO production and subsequent vasodilation of the vessel. The ratio of the average amplitude of the PAT signal over a one minute period starting one minute after cuff deflation (maximum pulse amplitude) divided by the average amplitude of the PAT signal over a 3.5 minute period before cuff inflation (baseline pulse amplitude) was calculated. The control arm was used to correct for confounding factors (room temperature, systemic changes). The result is expressed as the reactive hyperaemia index (RHI). Vascular measurements were performed as previously described. [9] To keep the focus on OS, detailed results of vascular measurements are described separately.

### Statistical analysis

Statistical analysis was performed using SPSS version 22.0 and GraphPad Prism version 7. Data are expressed as mean ± standard deviation (SD). Normality of continuous variables was evaluated using Kolmogorov-Smirnov test. Groups were compared using independent T-test, paired sample T-test and Mann-Whitney U test as appropriate. Fisher-exact test was used for comparison of categorical variables. Spearman and Pearson correlation coefficient was used for univariate correlation analysis as appropriate. A two-tailed p < 0.05 was considered significant.

## Results

### Patient characteristics

Characteristics of the three groups (HP, PE and NP) are summarized in Table 1. Groups were comparable regarding age, BMI and cardiovascular risk. Blood pressure was significantly different between groups. Due to urgency for delivery, only 17 of the 28 PE patients underwent vascular measurements. There was no significant difference between BMI in the NP population and BMI at 12weeks in HP (NP 23.0 ± 2.7 vs HP 24.0 ±4.2, p = 0.5).

**Table 1:** Patient characteristics.

	Preeclamptic pregnancy (n = 28)	Healthy pregnancy (n = 59)	Non-pregnant (n = 10)	p		
				PE vs HP	PE vs NP	HP vs NP
**Age (years)**	28.5 ± 3.9	30.2 ± 4.7	29.6 ± 4.1	0.27**		
**BMI 3rd trimester (kg/m2)**	30.2 ± 5.0	28.8 ± 4.2	22.3 ± 2.8	<0.0001*		
				0.99	<0.0001	<0.0001°
**SBP 3rd trimester (mmHg)**	160.2 ± 14.9	123.0 ± 9.2	122.9 ± 9.3	<0.0001*		
				<0.0001	<0.0001	>0.99
**DBP 3rd trimester (mmHg)**	96.0 ± 11.8	72.9 ± 8.2	73.8 ± 6.8	<0.0001*		
				<0.0001	<0.0001	>0.99
**Nulliparous (n)**	23 (82%)	31 (53%)	9 (90%)	0.004*		
				0.008	>0.99	0.07
**Gestation at delivery (weeks)**	32.0 ± 3.0	38.2 ± 2.2	/	<0.0001	/	/
**Smoking (n)**	0	0	0	/	/	/

Data are expressed as mean ± SD or as number of total (n). BMI = Body Mass Index, SBP = Systolic Blood Pressure, DBP = Diastolic Blood Pressure. PE = Preeclampsia, HP = Healthy Pregnancy, NP = Non-pregnant.

° There was no significant difference in BMI between NP and HP at 12 weeks (p = 0.50).

* Statistical analysis was performed using Kruskal-Wallis.

** Statistical analysis was performed using ANOVA.

### Oxidative stress and systemic inflammation in healthy pregnancy

**Fig 1:**
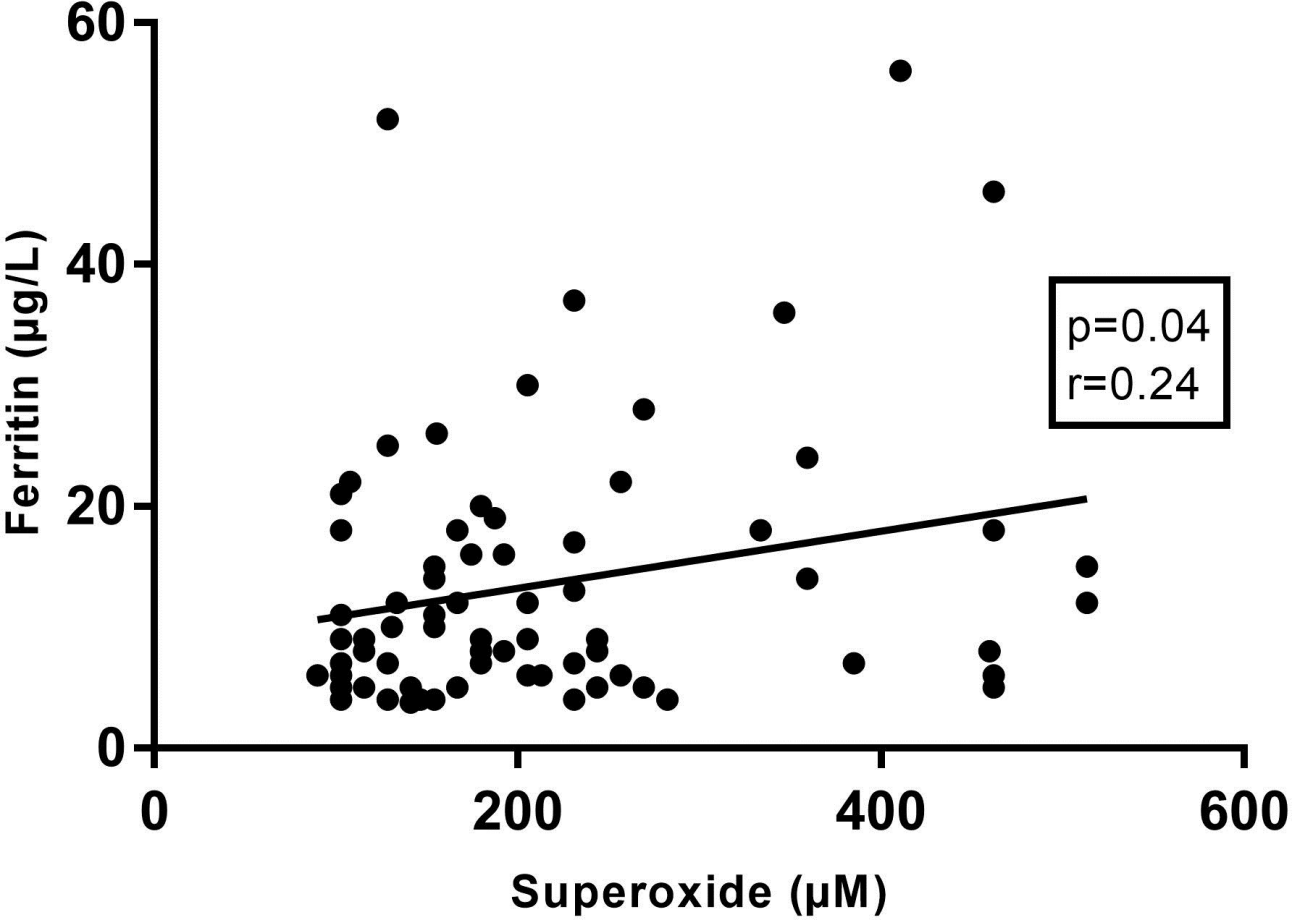
Correlation of superoxide concentrations with ferritin levels at 25 weeks of pregnancy. (r: pearson correlation coefficient).

During the course of a HP, NLR significantly increased (10 weeks 3.3±1.4 vs 25 weeks 4.7±1.8, p<0.001) caused by an increase in neutrophils (10 weeks 5.9±1.4 10^9^/L vs 25 weeks 7.2±1.9 10^9^/L, p<0.001) and a decrease in lymphocytes (10 weeks 1.9±0.7 10^9^/L vs 25 weeks 1.7±0.5 10^9^/L, p<0.001), while no difference was noted in MPV (10 weeks 8.4±1.2 fL vs 25 weeks 8.3±0.6 fL, p = 0.38). Superoxide concentrations remained stable during HP (10 weeks 197.1±73.0 μM vs 25 weeks 199.2±105.1 μM, p = 0.91) but were significantly higher compared to the NP controls (109.1±32.0, p<0.0001). Superoxide concentration at 25 weeks compared to 39 weeks (without labour) was not significantly different (25 weeks 208.3±131.1 μM vs 39 weeks 286.8±140.7 μM, p = 0.28), confirming a stable superoxide environment during the whole pregnancy. Iron (10 weeks 101.5±38.0 μg/dL vs 25 weeks 81.7±35.9 μg/dL, p<0.001) and ferritin (10 weeks 42.0±28.2 mg/L vs 25 weeks 15.3±10.9 μg/L, p<0.001) concentrations decreased, while transferrin concentrations (10 weeks 2.9±0.8 g/L vs 25 weeks 4.1±0.7 g/L, p = 0.007) increased with advancing pregnancy. At 25 weeks of pregnancy, there was a significant positive correlation between superoxide concentration and ferritin concentration. (Fig 1)

Regarding the blood samples taken at term, 60% of patients were in labour (a condition characterised by overt ischemia and reperfusion injury), resulting in a significant difference in NLR (labour 6.2±3.9 vs no labour 4.0±1.6, p<0.001) and superoxide concentrations (labour 409.8±105.8 μM vs no labour 303.9±119.8 μM, p = 0.03). NLR and superoxide during labour were positively correlated (p = 0.03).

### Oxidative stress and systemic inflammation in preeclampsia

In comparison with HP, OS and markers of systemic inflammation were much higher in PE. (Table 2) Regarding iron status, iron concentration and intracellular iron reserve (ferritin) were significantly higher in PE, while transferrin concentrations were not different. There was no significant correlation between superoxide and ferritin concentration in PE (p = 0.14), nor with other haematological parameters.

**Table 2:** Oxidative stress and systemic inflammation in PE vs HP.

	PE	HP	p
**Gestational age at blood sample**	32+3 weeks	25+3 weeks	>0.05
**Superoxide (μM)**	307.2 ± 108.7	199.2.0 ± 105.1	<0.0001
**NLR**	6.5 ± 4.7	4.7 ± 1.8	0.0003
**Neutrophils (10**^**9**^**/L)**	10.1 ± 5.1	7.2 ± 1.9	<0.0001
**Lymphocytes (10**^**9**^**/L)**	1.9 ± 0.7	1.6 ± 0.5	0.01
**MPV (fL)**	9.7 ± 1.8	8.3 ± 0.6	<0.0001
**Iron (μg/dL)**	115.3 ± 70.3	80.9 ± 44.1	0.0003
**Ferritin (μg/L)**	176.9 ± 325.2	13.5 ± 10.4	<0.0001
**Transferrin (g/L)**	3.8 ± 3.1	3.9 ± 3.0	0.82

Data are expressed as mean ± SD. NLR = Neutrophil to Lymphocyte ratio, MPV = Mean platelet volume.

### Correlation oxidative stress and arterial stiffness in healthy pregnancy

**Fig 2:**
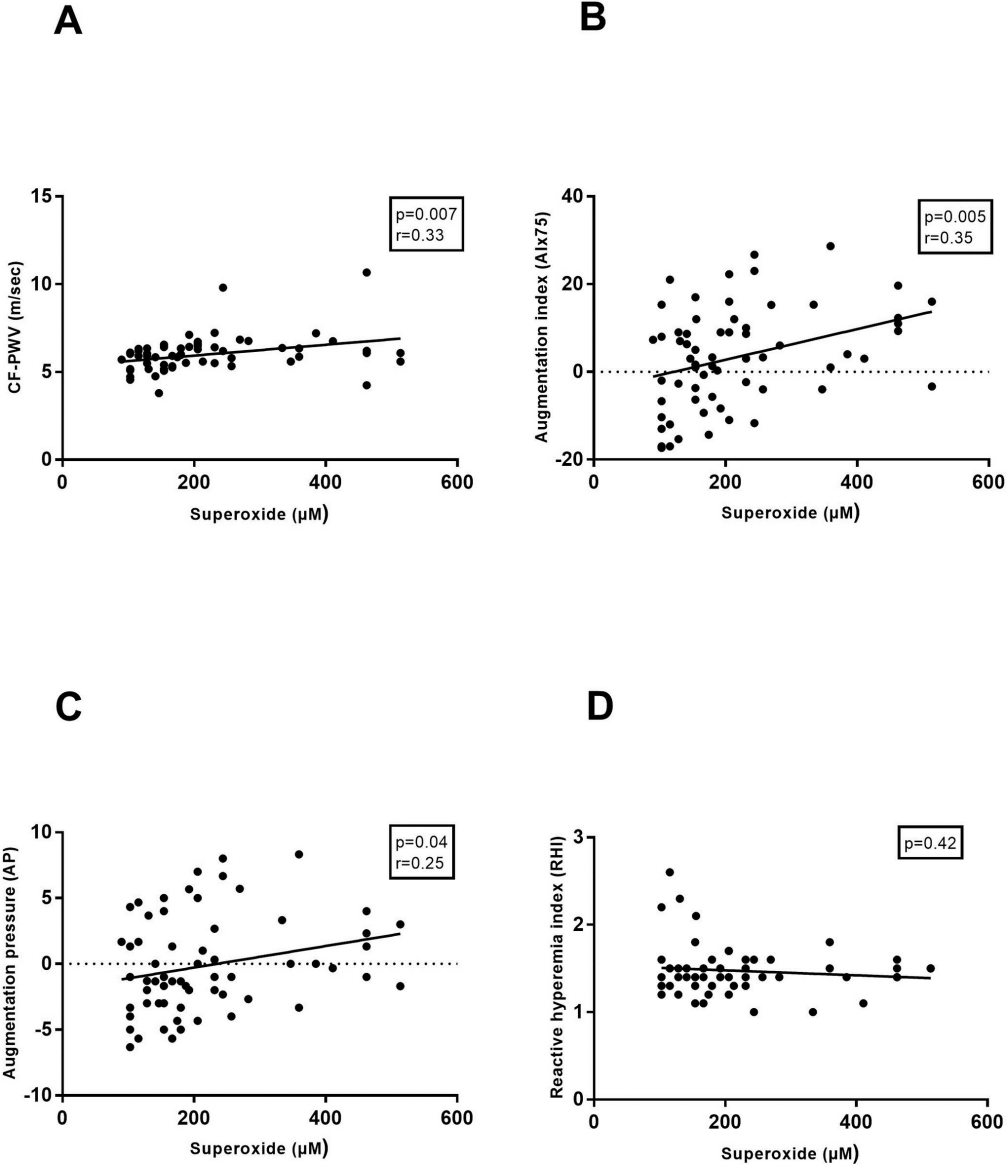
Correlation of superoxide concentrations with vascular function in the third trimester of a healthy pregnancy. Vascular function was assessed by carotid-femoral pulse wave velocity (CF-PWV (6.2±0.8), Fig 2A), augmentation index 75 (AIx75 (4.4±11.7), Fig 2B), augmentation pressure (AP (0.3±4.1), Fig 2C) and reactive hyperaemia index (RHI (1.6±0.4), Fig 2D). (r: pearson correlation coefficient).

In the HP group, superoxide concentrations at 25 weeks of HP correlated significantly with all markers of arterial stiffness (CF- PWV, AIx75 and AP) measured during the third trimester (mean 34+5weeks) (Fig 2A, 2B and 2C). There was, on the contrary, no significant correlation between superoxide and microvascular endothelial function (RHI) (Fig 2D).

### Correlation oxidative stress and microvascular endothelial function in preeclampsia

**Fig 3:**
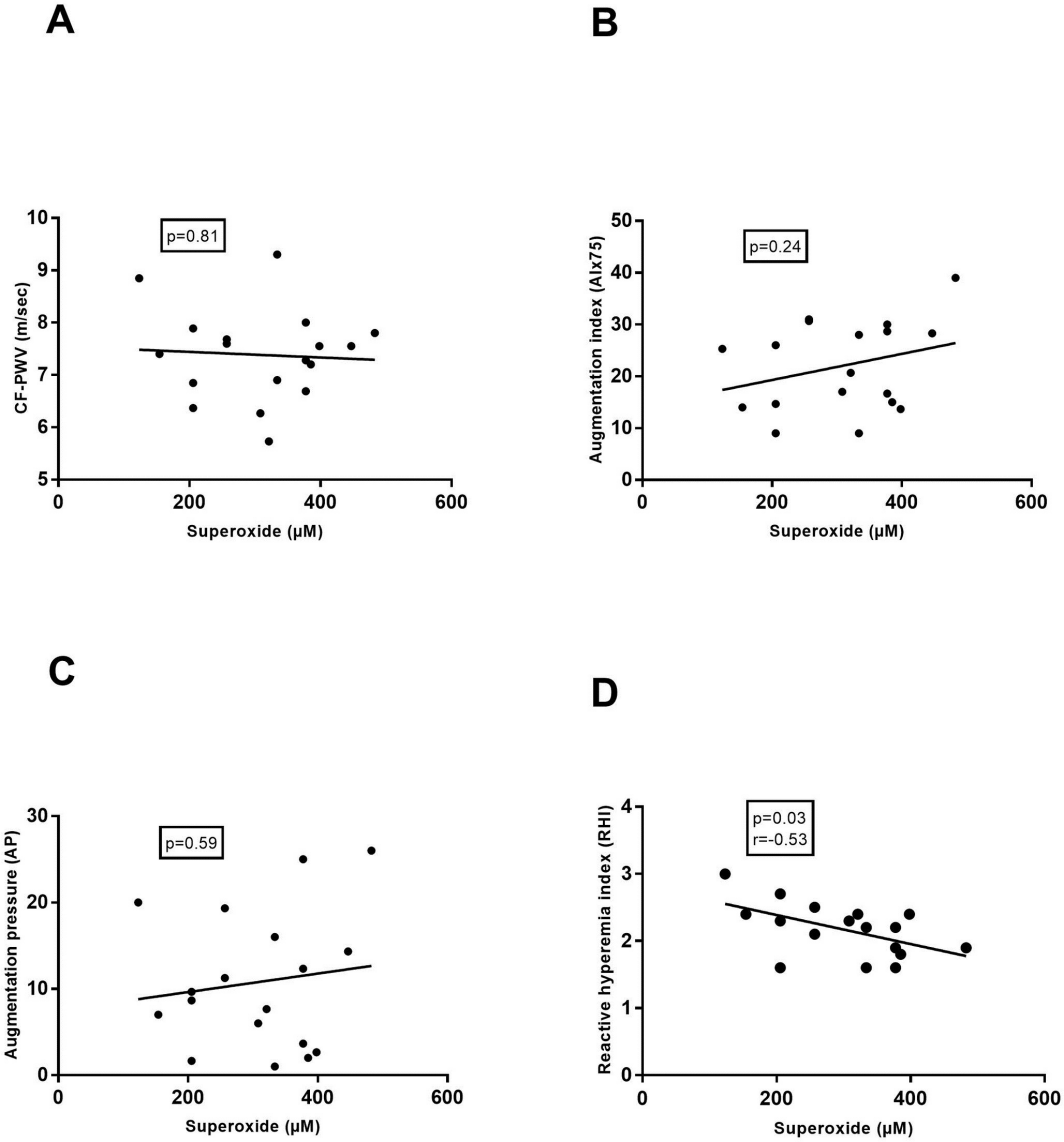
Correlation of superoxide concentrations with vascular function in preeclampsia. Vascular function was assessed by carotid-femoral pulse wave velocity (CF-PWV (7.6±0.9), Fig 2A), augmentation index 75 (AIx75 (24.0±9.6), Fig 2B), augmentation pressure (AP (11.8±7.8), Fig 2C) and reactive hyperaemia index (RHI (2.1±0.4), Fig 2D). (r: pearson correlation coefficient).

A significant negative correlation was observed between superoxide concentration and microvascular endothelial function (RHI) in PE (Fig 3D), while no correlation was found with arterial stiffness (CF- PWV, AIx75 and AP) (Fig 3A, 3B and 3C).

## Discussion

There are 4 major findings of this study on OS and systemic inflammation and their relationship with endothelial function and arterial stiffness in HP and PE.

First, consistent with previous studies, this study demonstrates that systemic inflammation increases with advancing pregnancy. During labour, a condition associated with brisk leucocytosis, a positive correlation was found between NLR and superoxide concentration, confirming the theory that activated blood cells and ischemia-reperfusion damage at the site of the placenta result in higher OS levels.

Second, OS appears to maintain stable concentrations during pregnancy, but is correlated to ferritin levels. Literature on the longitudinal course of OS during a HP is very scarce. An increased extracellular antioxidant status with advancing pregnancy and an increase in lipid peroxides in the second trimester have been described [27, 28], however our results imply a stable OS environment from the first trimester of pregnancy to term. Since placental OS is produced throughout pregnancy, is it probable that the antioxidant system acts in response to OS changes in order to maintain normal intercellular integrity and function in processes susceptible to OS in pregnancy. If this balance collapses, pregnancy complications might arise such as pregnancy-loss, intra-uterine growth restriction or PE. [3] Superoxide concentrations in the NP population are much lower compared to HP, proving once more the oxidative environment in a HP. Iron is abundantly present in the placenta and is an important co-factor in the formation of ROS. In the placenta, iron is stored in both ferritin and in transferrin bound to the transferrin receptor. [29] The placenta is a very oxygen-rich organ (starting from the second pregnancy trimester) and its abundant mitochondrial mass favours the production of ROS. Since iron is a necessary cofactor in the production of free radicals, iron excess results in acceleration of the formation of OS. On the contrary, iron deficiency results in defective mitochondrial function and mitochondrial DNA damage, with results in the release and leakage of ROS out of deficient mitochondria. [30, 31] Since both iron deficiency and iron excess result in free radical mitochondrial damage, iron supplementation is now only advisable for pregnant women with proven iron-deficiency anaemia. [30, 32] In this study, we found a positive correlation between superoxide concentration and ferritin concentration at 25 weeks of pregnancy, which confirms that an excess in iron supplementation can cause harm by augmenting the amount of OS. This finding has previously been described in another pregnancy complication, gestational diabetes mellitus. Research by Hininger et al. describes a high maternal iron status and iron supplementation in the non-anaemic pregnant population as a potential risk factor for the development of gestational diabetes mellitus and suggests that a ferritin level of >70 g/L may be associated with a higher risk of developing gestational diabetes. They proposed association of iron supplementation with vitamin E and antioxidant rich foods to prevent the formation of oxidative radicals associated with iron overload in pregnancy. [33, 34]

Third, PE is characterized by higher levels of superoxide, markers of systemic inflammation and iron status (Table 2) which is in line with previous literature. [3, 35, 36] Studies on OS in PE have mainly focussed on indirect biomarkers of OS or on antioxidants levels, while the methodology of our work is entirely different. In this study we were able to directly measure the amount of CM radical formed which is proven to correspond directly to the amount of superoxide. [18–21]We acknowledge that the concentration of the formed CM radical may not represent the total concentration of the superoxide radical anion which was present in the solution. However, their concentrations are directly related, allowing to obtain trends for the superoxide formation. [18, 19] Superoxide levels were clearly higher in PE and we hypothesize that this is induced by activated blood cells, the ischemic placenta, higher iron and ferritin levels and the inability of the antioxidant system to meet and neutralise the produced ROS. Recently, the ratio of neutrophil to lymphocyte has been proposed as a prognostic and predictive marker in several low-grade inflammation disease states, such as cancer, cardiac diseases and PE. [8, 37–40] Although lymphocyte levels were higher in our PE population, NLR remained significantly higher, endorsing once again the activation of peripheral blood cells in PE.

Lastly, this study innovatively approaches vascular function by affirming its direct relationship with the amount of OS present in peripheral blood. HP is associated with profound alterations in the maternal cardiovascular system. Due to a decrease in vascular resistance necessary to answer the higher needs of the growing foeto-placental unit, vascular stiffness during HP is lower compared to the NP state. [41, 42] In our HP study population we assessed arterial stiffness with applanation tonometry and although the results were in the normal range for a pregnant population, there was a significant positive relationship between OS concentration and vascular stiffness in the third pregnancy trimester. This finding might imply that when OS rises above a certain ‘harmful’ level, vascular damage arises resulting in vascular dysfunction and stiffness. [4, 5] On the contrary, in our PE population vascular stiffness was higher, yet no correlation was found between the amount of OS and vascular stiffness. A possible explanation could be that at a certain level of OS, vascular stiffness reaches a maximum, without increasing further with higher OS. Microvascular endothelial function on the other hand, is inversely correlated to superoxide concentration, evidenced by worse RHI results in PE patients with higher superoxide levels.

Despite these novel findings, our study has limitations. There was a significant difference in parity between the HP and PE group, which could influence our results. Furthermore, since blood samples at term were taken during labour in 60% of the cases, the sample size to compare blood parameters at 39 weeks without labour was small. Likewise, vascular measurements were performed in a subgroup of PE patients. Last, we did not record iron supplementation in our population, as a result there are no reliable data on the intake of iron-supplements. From previous published literature, we know that in our population approximately 76% of pregnant women take vitamin supplements, containing approximately 26mg of iron daily. [43] Since iron supplementation might be determinant in the superoxide production, interventional studies are planned in the future and exact monitoring and randomisation of iron intake will take place.

To the best of our knowledge, this is the first paper to compare superoxide concentrations between HP and PE directly measured with EPR and to correlate them with vascular function tests. The strengths of this study were the repeated measures of systemic inflammation and OS during HP and the finding that vascular function was correlated with the amount of OS in both HP and PE.

## Conclusions

The results of this study allow us to conclude that during HP there is an increased but stable oxidative environment, which is correlated to ferritin concentration. If superoxide levels increase, there is an augmentation in arterial stiffness. In PE pregnancies, systemic inflammation and superoxide concentrations are higher and result in a deterioration of microvascular endothelial function. Together, these findings support the hypothesis that vascular function is directly linked to the amount of OS, suggesting an important place for antioxidant treatment in the prevention on PE. Future research is imperative to investigate a broader spectrum of ROS in PE and to explore the effect of antioxidant therapy on vascular function.
